# Adoption of prophylactic cranial irradiation (PCI) for extensive stage small cell lung cancer (ES-SCLC): a population based outcome study

**DOI:** 10.1186/s13014-018-1184-x

**Published:** 2018-12-14

**Authors:** Yu Yang Soon, Huili Zheng, Shaun Zhirui Ho, Wee Yao Koh, Cheng Nang Leong, Jeremy Chee Seong Tey, Balamurugan Vellayappan, Swee Peng Yap, Ivan Weng Keong Tham, Kam Weng Fong

**Affiliations:** 1 Department of Radiation Oncology, National University Cancer Institute, 1E Kent Ridge Road, NUHS Tower Block, Level 7, Singapore, 119228 Singapore; 2 National University Hospital, 1E Kent Ridge Road, NUHS Tower Block, Level 7, Singapore, 119228 Singapore; 3 National University Health System, 1E Kent Ridge Road, NUHS Tower Block, Level 7, Singapore, 119228 Singapore; 4 National University of Singapore, 1E Kent Ridge Road, NUHS Tower Block, Level 7, Singapore, 119228 Singapore; 5 National Registry of Disease Office, Research & Surveillance Division, Health Promotion Board, Singapore, Singapore; 6 Division of Radiation Oncology, National Cancer Center Singapore, Singapore, Singapore

**Keywords:** Small cell lung cancer, Prophylactic cranial irradiation, Radiotherapy

## Abstract

**Background:**

The survival benefit of PCI in ES-SCLC reported by a European randomized trial (RCT) in 2007 was not replicated by a Japanese RCT published in 2017. This study aimed to evaluate the uptake of PCI before and after publication of the European RCT and its association with survival in ES-SCLC.

**Methods:**

We identified eligible patients in the only two Singapore national cancer centres from 2003 to 2010. We linked their electronic medical records to the national death registry. We described the utilization of PCI in patients diagnosed from 2003 to 2006 (pre-adoption cohort) with patients diagnosed from 2007 to 2010 (post-adoption cohort). We performed univariable and multivariable Cox regression analysis to assess the association between PCI and survival.

**Results:**

We identified 224 patients with ES-SCLC with no brain metastases. Among the 71 patients who had at least stable disease after first line chemotherapy, there was an increase in the use of PCI from the period 2007 to 2010 compared with 2003 to 2006 (32% versus 10%, *P* = 0.044). PCI was associated with improved OS (hazard ratio 0.22, 95% CI 0.10 to 0.47, *P* < 0.001) compared to no PCI in the multivariable analysis.

**Conclusion:**

There was an increase in the adoption of PCI for ES-SCLC since 2007. PCI was associated with improved survival in patients who did not have mandatory MRI brain imaging prior to PCI and had stable disease or better after first line chemotherapy, suggesting that the results of the European RCT are reproducible in the real-world practice.

## Introduction

Small cell lung carcinoma (SCLC) accounts for 13% of all newly diagnosed lung cancers, with 56% of new cases presenting as extensive stage disease (ES-SCLC) [1]. Approximately 18% of patients with SCLC present with brain metastases [2]. Patients with SCLC are at high risk of developing brain metastases with the probability of up to 80% at two years from diagnosis [3].

Prophylactic cranial irradiation (PCI) has been shown to improve overall survival (OS) in patients with ES-SCLC who had a response to first line chemotherapy in the European Organization for Research and Treatment of Cancer (EORTC) 08993–22,993 study [4]. This study extended the indication for PCI in SCLC not only for limited stage disease, but also for extensive stage disease. However, due to the concerns about the lack of magnetic resonance imaging (MRI) of the brain prior to enrolment and various chemotherapy regimens and PCI doses in the EORTC trial, the Japanese have conducted a randomized trial including 224 patients to re-assess the role of PCI in patients with ES-SCLC who responded to platinum based doublet chemotherapy and no brain metastases on magnetic resonance imaging (MRI) [5]. The results of this Japanese trial was published in 2017 which showed that PCI did not improve OS compared with regular three monthly brain MRI surveillance, questioning the survival benefits of PCI reported in the EORTC trial.

While randomized clinical trials provide the best quality evidence to guide clinical practice, there can be concerns regarding their external validity i.e. whether these interventions can be broadly applied to the general population [6]. Population-based outcome studies are increasingly recognized as important in providing insight into practice patterns and the impact of a change in practice and / or policy on health related outcomes. These studies allow the investigators to elucidate the uptake, safety and outcomes of novel medical therapies in the real world.

We undertook this retrospective study to evaluate the uptake of PCI for ES-SCLC and to assess its impact on overall survival before and after the publication of EORTC 08993–22,993 study [4].

## Methods

### Study design

This was an institutional review board-approved retrospective cohort study.

### Study population

Patients with histologically confirmed SCLC diagnosed between January 2003 and December 2010 were re-staged according to the American Joint Committee of Cancer seventh edition criteria [7]. Those with Stage IV SCLC (i.e. extensive stage) with no brain metastases treated with palliative intent in the only two Singapore national cancer centers (National University Cancer Institute, Singapore and National Cancer Center Singapore) were included. All patients were staged with computed tomography (CT) of thorax and abdomen. The use of brain imaging (MRI) or contrast-enhanced CT) or positron emission tomography (PET)-CT scans were not mandated. The time period 2003 to 2010 was specifically chosen to allow us to capture a cohort of ES-SCLC who would be managed similarly to the population in the EORTC trial [4].

The pre-adoption cohort included patients diagnosed with SCLC between January 2003 to December 2006, while the post-adoption cohort included patients diagnosed with SCLC between January 2007 and December 2010. We defined the cohorts in this fashion because the EORTC trial was published in 2007. PCI was administered with the use of two opposed lateral fields with 6 MV photons with varying dose / fractionation schedules ranging from 25 Gy in 10 fractions, 30 Gy in 10 fractions and 30.6 Gy in 17 fractions. Palliative thoracic radiation therapy was delivered using conformal radiation therapy techniques with varying dose / fractionation schedules ranging from 20 Gy in 5 fractions, 30 Gy in 10 fractions, 36 Gy in 12 fractions and 40 Gy in 16 fractions.

### Co-variates

Clinical data was collected from the institutional electronic medical records. Age at diagnosis (< 70 and ≥ 70 years old), gender, Eastern Cooperative Oncology Group (ECOG) performance status (PS) (1 and 2–4), smoking status (smoker, ex-smoker and lifetime non-smoker), modified Charlson’s comorbidity index, excluding diagnosis of SCLC (0, 1–2 and ≥ 3) [8], brain imaging at diagnosis, received first line chemotherapy, number of cycles of first line chemotherapy (1–3 and 4–6), use of platinum based first line chemotherapy, second line chemotherapy, more than two lines of chemotherapy, PCI, thoracic radiation, whole brain radiation and skeletal radiation were analyzed as categorical variables. The response to first line chemotherapy was assessed using the modified Response Evaluation Criteria in Solid Tumors within eight weeks of last cycle of chemotherapy [9].

### Endpoints

The unique national identification number assigned to all Singapore residents was used to link the study’s cohorts to the national death registry. The death registry contains information on the date and cause of death for all Singapore residents.

### Statistical analysis

Frequency with percentage was used to describe the baseline characteristics of the overall study population as well as a subset of population who had at least stable disease after first line chemotherapy. The differences in the baseline characteristics between the pre and post-adoption cohorts was analyzed using the exact Fisher test or Chi-square test where appropriate. Time to death was measured from date of diagnosis of SCLC to death from any cause. The Kaplan-Meier curves was used to describe the time to event data for the pre and post-adoption cohorts. The log-rank test was used to compare the time to event intervals between the pre and post adoption cohorts. Univariable Cox regression analysis was used to determine the association between death and baseline characteristics. Variables which were statistically significant in the univariable analysis as well as variables which are expected to affect survival, namely age, performance status, use of whole brain radiation were included in the multivariable analysis regardless of their statistical significance findings in the univariable analysis. A sensitivity analysis including only patients who had brain imaging was performed to investigate if the use of brain imaging at diagnosis is a potential effect modifier on the relationship between use of PCI and survival outcome. For all analyses, two-sided *P* values of less than 0.05 were considered statistically significant. Analyses were performed using STATA (version 13.0, StataCorp).

## Results

### Baseline characteristics of study population

We identified 224 patients with ES-SCLC without brain metastases. 71 patients had stable disease or better with first line chemotherapy. The baseline characteristics of these 71 patients were summarized in Table 1. The date of last censorship for the pre and post-adoption cohorts was 30th June 2010 and 30th June 2014 respectively. Slightly more than half of the patients were aged 70 years or above. Majority of the patients were men and current smokers. More than 80% of the patients had Charlson’s co-morbidity index score of 0–2. More than half of the patients had brain imaging for staging at diagnosis. Less than half of the patients received radiotherapy treatment including PCI, palliative thoracic, therapeutic whole brain or skeletal radiation.

**Table 1:** Baseline characteristics of patients who had at least stable disease after first line chemotherapy

Characteristic	2003–2006 (*n* = 30)		2007–2010 (*n* = 41)		*P* value
	No. of patients	%	No. of patients	%	
Sociodemographic and clinical characteristics					
Age at diagnosis, years					
< 70	14	46.7	20	48.8	0.860
≥ 70	16	53.3	21	51.2	
Gender					
Male	25	83.3	38	92.7	0.269
Female	5	16.7	3	7.3	
ECOG PS					
1	21	70.0	35	85.4	0.117
2–4	9	30.0	6	14.6	
Smoking status					
Lifetime non-smoker	1	3.3	1	2.4	0.999
Ex-smoker	9	30.0	13	31.7	
Smoker	20	66.7	27	65.9	
Charlson’s co-morbidity index					
0	16	53.3	19	46.3	0.738
1–2	12	40.0	20	48.8	
≥ 3	2	6.7	2	4.9	
Had brain imaging at diagnosis	17	56.7	27	65.9	0.431
Chemotherapy treatment characteristics					
No. of cycles of first line chemotherapy					
1–3	5	16.7	4	9.8	0.493
4–6	25	83.3	34	82.9	
Not reported	0	0.0	3	7.3	
Received Platinum based first line chemotherapy	28	93.3	38	92.7	0.999
Received second line chemotherapy	5	16.7	20	48.8	0.005
Received more than two lines of chemotherapy	1	3.3	6	14.6	0.226
Radiotherapy treatment characteristics					
Received prophylactic cranial irradiation	3	10.0	13	31.7	0.044
Received thoracic radiation	9	33.0	16	39.0	0.432
Received whole brain radiation	12	40.0	17	41.5	0.901
Received skeletal radiation	5	16.7	4	9.8	0.479

**Fig. 1:**
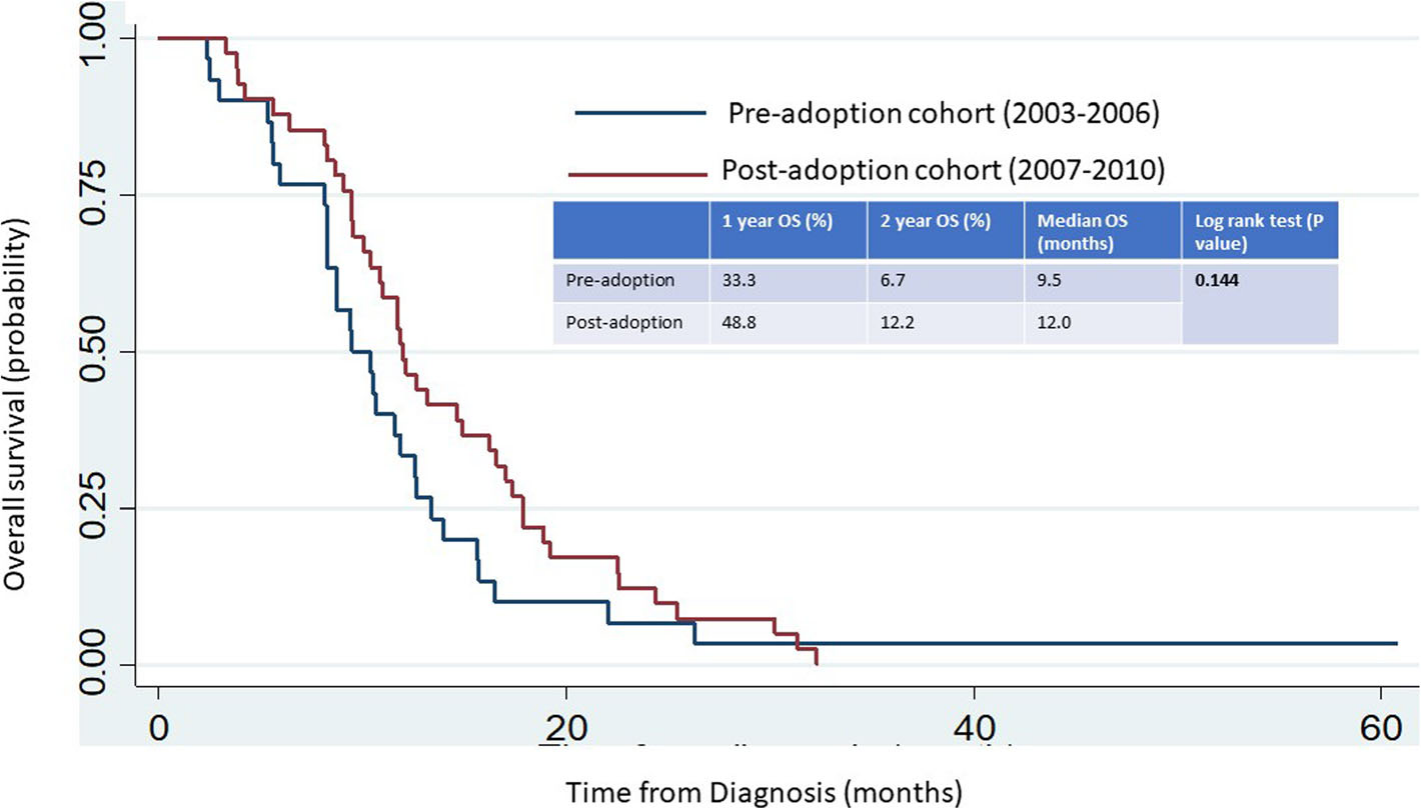
Overall survival outcomes of patients with ES-SCLC who had at least stable disease or better after initial chemotherapy

There were statistically significantly greater percentage of patients who received second line chemotherapy (49% vs 17%, *P* = 0.005) and PCI (32% vs 10%, *P* = 0.044) (Table 1) in the post-adoption than the pre-adoption cohort. There was no statistically significant difference in the overall survival outcome between the pre and post-adoption cohorts (log rank *P* = 0.144) (Fig. 1).

### Univariable analysis on factors associated with overall survival

There was a total of 70 deaths among patients who had at least stable disease after first line chemotherapy. Univariable analysis showed that number of cycles of first line chemotherapy and use of PCI, palliative thoracic and skeletal radiation are associated with survival outcome (Table 2). The use of PCI was also found to be significantly associated with overall survival outcome when the analysis was limited to patients who had brain imaging at diagnosis (Table 3).

**Table 2:** Univariable and multivariable Cox regression analysis: Characteristics associated with overall survival for patients who had at least stable disease after first line chemotherapy

Characteristic	Univariable analysis			Multivariable analysis		
	HR	95% CI	*P* value	HR	95% CI	*P* value
Sociodemographic and clinical characteristics						
Age at diagnosis, years						
≥ 70 (vs < 70)	1.17	0.73–1.88	0.506	1.02	0.60–1.73	0.952
Gender						
Male (vs Female)	0.47	0.22–1.01	0.053			
ECOG PS						
2–4 (vs 1)	1.76	0.98–3.14	0.057	2.53	1.12–5.70	**0.025**
Smoking status						
Ex-smoker (vs Lifetime non-smoker)	3.37	0.78–14.46	0.103			
Smoker (vs Lifetime non-smoker)	2.07	0.50–8.62	0.316			
Charlson’s co-morbidity index						
1–2 (vs 0)	1.24	0.75–2.06	0.400			
≥ 3 (vs 0)	2.24	0.77–6.54	0.139			
Had brain imaging at diagnosis	1.28	0.78–2.10	0.334			
Chemotherapy treatment characteristics						
No. of cycles of first line chemotherapy						
4–6 (vs 1–3)	0.36	0.17–0.74	**0.005**	0.21	0.09–0.50	**< 0.001**
Received Platinum based first line chemotherapy	0.81	0.32–2.02	0.645			
Received second line chemotherapy	0.64	0.39–1.05	0.077			
Received more than two lines of chemotherapy	0.50	0.22–1.11	0.087			
Radiotherapy treatment characteristics						
Received prophylactic cranial irradiation	0.35	0.19–0.65	**0.001**	0.22	0.10–0.47	**< 0.001**
Received thoracic radiation	0.52	0.31–0.87	**0.014**	0.49	0.28–0.86	**0.013**
Received whole brain radiation	0.92	0.57–1.49	0.742	0.32	0.17–0.59	**< 0.001**
Received skeletal radiation	2.28	1.09–4.76	**0.028**	0.83	0.33–2.13	0.704

Boldface data are value less than 0.05

**Table 3:** Univariable and multivariable Cox regression analysis: Characteristics associated with overall survival for patients who had brain imaging at diagnosis and at least stable disease after first line chemotherapy

Characteristic	Univariable analysis			Multivariable analysis		
	HR	95% CI	*P* value	HR	95% CI	*P* value
Sociodemographic and clinical characteristics						
Age at diagnosis, years						
≥ 70 (vs < 70)	1.08	0.59–1.97	0.812	1.33	0.66–2.70	0.428
Gender						
Male (vs Female)	0.20	0.07–0.57	**0.003**			
ECOG PS						
2–4 (vs 1)	2.39	1.17–4.87	**0.017**	2.80	1.08–7.23	**0.034**
Smoking status^a^						
Smoker (vs Lifetime non-smoker)	1.35	0.72–2.54	0.343			
Charlson’s co-morbidity index						
1–2 (vs 0)	1.49	0.77–2.88	0.241			
≥ 3 (vs 0)	2.79	0.88–8.85	0.082			
Chemotherapy treatment characteristics						
No. of cycles of first line chemotherapy						
4–6 (vs 1–3)	0.37	0.17–0.79	**0.011**	0.31	0.12–0.83	**0.020**
Received Platinum based first line chemotherapy	0.27	0.08–0.93	**0.038**			
Received second line chemotherapy	0.48	0.25–0.92	**0.027**			
Received more than two lines of chemotherapy	0.38	0.13–1.11	0.076			
Radiotherapy treatment characteristics						
Received prophylactic cranial irradiation	0.37	0.17–0.77	**0.008**	0.11	0.04–0.32	**< 0.001**
Received thoracic radiation	0.55	0.30–1.03	0.063	0.47	0.22–0.98	**0.045**
Received whole brain radiation	0.77	0.42–1.41	0.395	0.18	0.08–0.43	**< 0.001**
Received skeletal radiation	2.06	0.88–4.85	0.097	0.50	0.16–1.62	0.248

^a^no ex-smoker died prior to censor date

Boldface data are value less than 0.05

### Multivariable analysis on factors associated with overall survival

Among the patients who had at least stable disease after first line chemotherapy, multivariable analysis showed that patients with ECOG performance status as no symptom (HR 0.36, 95% CI 0.14–0.92, *P* = 0.034), those who received more than three cycles of first line of chemotherapy (HR 0.31, 95% CI 0.12–0.83, *P* = 0.020), those who received PCI (HR 0.11, 95% CI 0.04–0.32, *P* < 0.001), those who received thoracic radiation (HR 0.47, 95% CI 0.22–0.98, *P* = 0.045) and those who received whole brain radiation (HR 0.18, 95% CI 0.08–0.43, P < 0.001) had better overall survival outcomes (Table 2). When the multivariable analysis was limited to patients who had brain imaging at diagnosis, we also observed that the use of PCI was associated with statistically significant improvement in overall survival (HR 0.11, 95% CI 0.04–0.32, *P* value < 0.001) adjusting for age, ECOG performance status, the number of cycles of first line chemotherapy, use of whole brain, thoracic and skeletal radiation therapy (Table 3).

## Discussion

This study showed that there was an increase in the adoption of PCI for ES-SCLC since the publication of the EORTC trial in 2007 [4], and PCI was associated with improved overall survival among patients who had at least stable disease or better after first line chemotherapy.

The results of this study are consistent with the EORTC trial and other recent published retrospective cohort studies [10, 11]. Chen et al. evaluated 204 patients with ES-SCLC who had any response to initial chemotherapy and had MRI or CT brain imaging to exclude brain metastases prior to the start of initial chemotherapy [10]. They found that PCI significantly improved median OS from 12.6 to 16.5 months with HR 0.63, 95%CI 0.41–0.96, *P* = 0.033), adjusting for tumor load, number of metastatic sites and liver metastases in the multivariable Cox regression analysis. Bang and colleagues reviewed 155 patients with ES-SCLC who had at least partial response to initial chemotherapy and MRI or CT brain imaging at diagnosis [11]. They observed that PCI was associated with improved overall survival (HR 0.55, 95% CI 0.39–0.77, *P* = 0.0005) adjusting for age, gender, ECOG performance status, smoking history, presence of extra-thoracic metastases, types of post-chemotherapy brain imaging, types of platinum chemotherapy regimens, number of cycles of chemotherapy and types of response to chemotherapy in the multivariable Cox regression analyses. Like EORTC trial, for patients who did not receive PCI in these studies, including ours, active MRI brain imaging surveillance was not mandatory.

We observed that there was an non-statistically significant improvement in the unadjusted one-year (49% vs 33%) and two-year overall survival rate (12% vs 7%) in the post-adoption cohort compared to the pre-adoption cohort. The improvement in the survival rates may be due to more patients in the post-adoption cohort group receiving PCI and second line chemotherapy. The lack of statistical significance is possibly due to the small sample size of this study.

When we limit the analysis to patients who had brain imaging at diagnosis either with CT or MRI, we continue to observe that the use of PCI was associated with improved overall survival in both uni and multivariable analysis. There could be a few explanations for this observation. Firstly it is possible that these patients may harbour intra-cranial micro-metastatic disease prior to PCI as brain imaging was not mandatory prior to PCI and CT brain imaging at diagnosis may not have picked at these micro-metastatic disease. Potentially, the survival benefit of PCI in these patients may be due to treating these micro-metastases early. Secondly, for patients who did not receive PCI, routine MRI brain imaging surveillance was not performed. These patients may not have received salvage intracranial therapy promptly, leading to worse survival outcomes compared to patients who received PCI. As shown in the Japanese RCT comparing PCI with active MRI brain imaging surveillance plus prompt salvage intracranial treatment, there was no overall survival difference between the two groups [5].

Interestingly, we also observed that the use of palliative thoracic radiation therapy was associated with improvement in overall survival as well. We did not differentiate whether thoracic radiation therapy was used to consolidate chemotherapy response or to palliate thoracic disease after progression of first line chemotherapy. Nonetheless, this finding is consistent with the secondary analysis of the CREST randomized trial which showed that thoracic radiation therapy improved overall survival in patients with ES-SCLC who had residual intra-thoracic disease after first line chemotherapy [12, 13]. Currently, the role of consolidation thoracic radiation therapy in ES-SCLC is unclear with conflicting findings from randomized trials. Although the CREST trial did not demonstrate a statistically significant survival benefit with the use of consolidation thoracic radiation therapy in its primary analysis, its secondary analysis showed that consolidation thoracic radiation therapy significantly improved the two-year OS from 3 to 13% which was an important and clinically relevant finding [12]. A randomized phase 2 trial from the Radiation Therapy Oncology Group with similar design to the CREST trial also failed to demonstrate that consolidation thoracic radiation can improve overall survival in patients with ES-SCLC [14].

The strengths of this study are as follows. Firstly, we used the national death registry to ensure a complete follow up of our study population, resulting in a high level of consistency and accuracy for the analysis of the overall survival endpoint. Secondly, we have chosen a time period of 2003 to 2010 where clinical management was likely similar to that of the EORTC trial. Lastly, the results of this study were consistent with other retrospective cohort studies [10, 11].

There are some limitations to this study. Firstly, the sample size of this study is smaller compared to the other retrospective cohort studies [10, 11], although we did observe similar results. Secondly, the use of PCI remained fairly low despite an increase in its use post publication of the EORTC trial [4]. Thirdly, MRI brain surveillance was not routinely performed for patients who did not receive PCI, thus we were unable to determine if that will affect the survival benefit of PCI.

The implication of this study is that the results of the EORTC trial is reproducible in the real-world setting where MRI brain imaging are not routinely use to stage the disease at diagnosis or monitor intracranial disease progression. In clinical situation where patients with ES-SCLC with at least stable disease post initial chemotherapy decline regular active MRI brain imaging surveillance, PCI should be recommended.

## Conclusion

In conclusion, our study showed that there was an increase in the utilization of PCI post publication of the EORTC trial and PCI was associated with improved survival in patients with at least stable disease post initial chemotherapy. This suggests that the results of the EORTC trial are reproducible in the real-world settings particularly when MRI brain imaging was not routinely used for evaluation of the intracranial disease. Future research is warranted to confirm the findings of the Japanese RCT [5].

## Abbreviations

ECOG: Eastern Cooperative Oncology Group
EORTC: European Organization for Research and Treatment of Cancer
ES-SCLC: Extensive stage small-cell lung cancer
MRI: Magnetic resonance imaging
OS: Overall survival
PCI: Prophylactic cranial irradiation
PET-CT: Positron emission tomography-CT
SCLC: Small-cell lung cancer
